# Ionizing Radiation and Childhood Leukemia

**DOI:** 10.1289/ehp.10080

**Published:** 2007-08

**Authors:** Abel Russ

**Affiliations:** George Perkins Marsh Institute, Worcester, Massachusetts, E-mail: abelruss@riseup.net

I read with interest the recent review by Belson et al. (2007) on childhood leukemia, particularly the sections dealing with radiation exposure. Like the authors, I believe that ionizing radiation is strongly associated with childhood acute leukemia. I would like to point out that several critical pieces of information were overlooked; these support stronger and more meaningful conclusions.

Although atomic bomb survivors offer the clearest evidence of leukemia risk after childhood exposures to ionizing radiation, studies of children exposed to fallout in other contexts should not be downplayed. Belson et al. (2007) stated that “radiation exposure secondary to the Chernobyl accident has not been shown to increase the risk of leukemia in children who were exposed after birth …,” but they failed to mention the case–control study of Noshchenko et al. (2002), which found significant increases in childhood and acute leukemias in association with estimated childhood exposures. Children living downwind of the Nevada Test Site have also shown a significant increase in leukemia related to estimated fallout exposure (Stevens et al. 1990).

*In utero* exposure to ionizing radiation has been a known causal factor for childhood cancer for > 50 years. Although Belson et al. (2007) stated that the lack of evidence for a childhood leukemia risk among atomic bomb survivors constitutes the “most notable reason for doubt of a true association,” they overlooked the reviews of Wakeford and Little (2002, 2003); these authors demonstrated that the highly uncertain atomic bomb survivor data are statistically compatible with the robust set of data found in the Oxford Survey of Childhood Cancers and related X-ray exposure cohorts. There is no valid reason to doubt this association at present.

The association between preconception paternal irradiation (PPI) and childhood leukemia has always been controversial. Two of the major objections to the “Gardner hypothesis,” as Belson et al. (2007) pointed out, have been mixed evidence from studies of radiation-exposed fathers and a lack of positive evidence in the children of the atomic bomb survivors. Regarding the first objection, Belson et al. overlooked the two largest studies of the children of radiation workers. Draper et al. (1997) conducted a UK-wide case–control study of childhood cancers in relation to paternal radiation exposure. This study showed, based on > 13,000 cases not included in the study of Gardner et al. (1990), that children with leukemia or non-Hodgkin lymphoma were significantly more likely than controls to have fathers who were radiation workers. Dickinson and Parker (2002) conducted a cohort study of > 250,000 births in Cumbria, England, including the cases of Gardner et al. (1990), and found a significant 2-fold increase in the risk of leukemia and non-Hodgkin lymphoma among the children of radiation workers. These and other studies, taken together, give statistical support to the idea that paternal radiation work is a risk factor for childhood leukemia.

When interpreting the evidence for a PPI effect in atomic bomb survivors, it is important to consider what is known about potential mechanisms. As reviewed by Niwa (2003), Nomura (2003), and others, animal studies have consistently demonstrated that PPI can cause or increase the susceptibility to leukemia in offspring. In addition to fascinating evidence of postconception genomic instability after preconception exposure, many studies suggest that there may a window of sensitivity corresponding to post-meiotic stages of spermatogenesis; in humans, this would mean the few months leading up to conception (Adler 1996). Of the roughly 30,000 children of atomic bomb survivors, only about 2% were conceived in the 6 months after the bombings. Based on the spontaneous leukemia rate reported by Yoshimoto (1990), the expected number of spontaneous cases in this subcohort would be < 1, and an excess on the order suggested by the radiation worker studies would not be statistically apparent. For this and other reasons, the atomic bomb survivors may not be an appropriate comparison group.

To summarize, it is not unreasonable to observe that the weight of evidence generated to date supports the idea that preconception, prenatal, and postnatal exposures to ionizing radiation are all risk factors for childhood leukemia.

## Editor’s note

In accordance with journal policy, Belson et al. were asked whether they wanted to respond to this letter, but they chose not to do so.
